# Concerns, Beliefs and Attitudes of Pharmacists About Medical Cannabis Use in Poland

**DOI:** 10.3390/healthcare13212657

**Published:** 2025-10-22

**Authors:** Piotr Merks, Jameason Cameron, Justyna Kazmierczak, Artur Białkowski, Dariusz Świetlik, Mariola Borowska, Waldemar Wierzba, Urszula Bołkun-Skórnicka, Daniel Śliż, Eliza Blicharska, Jarosław Fedorowski, Regis Vaillancourt, Urszula Religioni

**Affiliations:** 1 Faculty of Medicine, Collegium Medicum, Cardinal Stefan Wyszyński University, 01-815 Warszawa, Poland; piotrmerks@googlemail.com (P.M.); regisvaillancourt@hotmail.com (R.V.); 2 Children’s Hospital of Eastern Ontario (CHEO), 401 Smyth Rd., Ottawa, ON K1H 5B2, Canada; jcameron@cheo.on.ca; 3 Zdrowit sp. z o.o., Pharmacy Chain, 41-940 Piekary Śląskie, Poland; justynamichner@gmail.com; 4 School of Public Health, Centre of Postgraduate Medical Education of Warsaw, 01-826 Warsaw, Poland; artur@bialkowscy.eu (A.B.); urszula.bolkun-skornicka@cmkp.edu.pl (U.B.-S.); urszula.religioni@gmail.com (U.R.); 5 Department of Biostatistics and Neural Networks, Medical University of Gdansk, 81-519 Gdynia, Poland; dariusz.swietlik@gumed.edu.pl; 6 Department of Cancer Epidemiology and Primary Prevention, Maria Sklodowska-Curie National Research Institute of Oncology, 02-781 Warsaw, Poland; 7 Department of Digital Medicine, Implementation and Innovation, National Medical Institute of the MSWiA, 02-507 Warsaw, Poland; waldemar.wierzba@pimmswia.gov.pl; 8 III Clinic of Internal Medicine and Cardiology, Medical University of Warsaw, 02-091 Warsaw, Poland; daniel.sliz@wum.edu.pl; 9 Department of Pathobiochemistry and Interdisciplinary Applications of Ion Chromatography, Medical University of Lublin, 1 Chodźki St., 20-093 Lublin, Poland; eliza.blicharska@umlub.pl; 10 Larner College of Medicine, University of Vermont, Burlington, VT 05401, USA; jjf@pfsz.org; 11 Faculty of Health Sciences, Zamojski Academy, 22-400 Zamość, Poland

**Keywords:** medical marijuana, medical cannabis, cannabis attitudes and beliefs, pharmacist, Poland

## Abstract

Introduction: The global use of medical cannabis is steadily increasing. In Poland, medical cannabis was legalised in 2017; however, its use remains limited and not widely integrated into clinical practice. This study aimed to explore the attitudes, concerns, and beliefs of pharmacists and pharmacy students regarding the medical use of cannabis. Methods: A study was conducted in 2021 among 422 pharmacists and pharmacy students in Poland, primarily working in community pharmacies. Data were collected using a custom-designed questionnaire addressing beliefs, concerns, and professional experiences related to medical cannabis. Results: Nearly half of the respondents (48.9%) believe that cannabis should be used exclusively for medical purposes, while 47.6% support its use for both medical and recreational purposes following legalisation. A substantial majority (over 90%) consider cannabis effective for treating adults, with nearly 70% acknowledging its potential for treating children. Over 66% of respondents feel comfortable discussing medical cannabis with patients; however, fewer are confident in providing detailed advice about its use. The findings also highlight concerns about the long-term effects and potential legal implications of dispensing medical cannabis. Conclusions: Pharmacists and pharmacy students in Poland demonstrate openness to the medical use of cannabis and recognise its therapeutic potential. However, to enhance their ability to advise patients effectively, targeted educational initiatives are needed. These should focus on the clinical applications, safety, and long-term effects of cannabis, alongside strategies for addressing patient concerns and ensuring responsible usage.

## 1. Introduction

Medical cannabis was legalised in Poland in 2017 [1], making it the 12th European country to approve cannabis for medical purposes. This legislative change aimed to enable physicians to prescribe medical cannabis in a controlled and lawful manner for patients whose conditions fail to respond to standard pharmacotherapy or for whom conventional treatments are overly burdensome. The overarching goal was to improve patient health outcomes and quality of life through access to medical cannabis [2]. The first supplies of medical cannabis became available in Polish pharmacies in early 2019, requiring a prescription for dispensation. However, despite legalisation, medical cannabis remains available in only a limited number of pharmacies, and its application in clinical practice is still relatively rare. This limited uptake reflects supply-chain and regulatory constraints (import licencing and magistral compounding), the small number of pharmacies equipped to prepare cannabis magistrals, lack of clear clinical guidance and prescriber training, and high out-of-pocket costs due to the absence of reimbursement [3]. From a health-economics perspective, these barriers—together with non-reimbursed patient payments—may dampen demand despite potential downstream savings associated with pharmacist-delivered services (fewer medication errors, fewer hospitalisations) [while potential gains in quality of life remain underrealized [2].

A growing body of scientific evidence highlights the clinical efficacy of cannabis in managing various diseases and symptoms. It has shown therapeutic potential in alleviating pain [4,5], mitigating nausea and vomiting [6], addressing symptoms of multiple sclerosis [7], improving sleep disorders [8], and aiding in the management of conditions such as cancer [9], epilepsy [10], and post-traumatic stress disorder [11]. Moreover, studies underscore the role of medical cannabis in enhancing patients’ quality of life [12]. However, not all research confirms these benefits; some studies have reported a lack of significant clinical effects from cannabis use in medical settings [13,14].

Globally, the number of patients using medical cannabis has steadily increased, reflecting its growing acceptance as a treatment option [15]. Pharmacists, as central figures in the safe and effective management of pharmacotherapy, are frequently consulted by both patients and prescribing physicians about the use of medical cannabis for various conditions. The accessibility and expertise of pharmacists can significantly influence treatment outcomes [16,17]. Furthermore, robust evidence supports the role of pharmaceutical services, including patient counselling, in reducing medication errors, preventing long-term health complications, and decreasing hospitalisations, which ultimately reduces healthcare costs on a global scale [18,19]. Nevertheless, limited research and insufficient education about the medical use of cannabis during academic training may shape healthcare professionals’ attitudes toward its use [20]. Given the critical role of pharmacists in patient care, medication safety, and public health education [21], understanding their perspectives on medical cannabis is essential to optimising its integration into healthcare systems.

This study aims to assess the attitudes, concerns, and beliefs of pharmacists and pharmacy students regarding the use of medical cannabis. Additionally, it seeks to explore differences in perceptions between these two groups to better understand the factors influencing their acceptance and application of medical cannabis in professional practice. By providing contemporary, Poland-specific data on perceived indications, benefits/risks, counselling comfort, and legal concerns, this study addresses a documented evidence gap and informs priorities for education, guidance, and policy relevant to clinical practice and health-economic decision-making.

## 2. Material and Methods

### 2.1. Study Design

The study was conducted in 2021 and targeted pharmacists and pharmacy students in Poland. Inclusion criteria included:

- Masters of Pharmacy graduates with a valid license to practice their profession, working in community pharmacies, hospital pharmacies/hospital pharmacy departments, healthcare institutions, or other places related to the practice of their profession;
- Students of a uniform Master’s degree programme in pharmacy (accredited faculties), of legal age.

Participants were recruited through a survey link shared on social media and via pharmacist organisations in Poland (including the Trade Union of Pharmaceutical Workers, ZZPF), which distributed the survey to their members. The survey was open from September to December 2021 and was disseminated via two social media platforms (Facebook and Twitter) as well as organisational mailing/communication channels; for pharmacists, we estimate that organisational distributions reached approximately 3300 members. To limit participation by individuals outside the target population, an eligibility screener at the start of the questionnaire required respondents to confirm their status as a pharmacist or pharmacy student in Poland; a negative response automatically terminated the survey. We also performed internal consistency checks (e.g., workplace, years of practice/study status). No personally identifying information (e.g., licence numbers) was collected. Participation in the study was voluntary, and respondents were informed about the anonymity of the survey and the use of the results exclusively for scientific purposes.

### 2.2. Research Instrument

The study used a custom-designed questionnaire that included questions about the respondents’ beliefs and concerns regarding the use of medical cannabis, as well as demographic data (gender, age, professional status, academic degree, years of practice, and workplace).

The tool was developed by an interdisciplinary team, including two clinical pharmacy experts and one public health expert, based on a literature review. Content validity was assessed by two independent pharmacy experts specialising in the discussed topic. A pilot study was conducted among a group of students (6) and pharmacists (4), which led to the clarification of some questions, although we consider the changes to be minor.

Most questions were assessed using a 5-point Likert scale, where 1 = Strongly Disagree, 2 = Disagree, 3 = Neither Agree nor Disagree, 4 = Agree, and 5 = Strongly Agree. For frequency-related questions, the following scale was used: 1 = Never, 2 = Seldom (less than twice a month), 3 = Sometimes (two to four times a month), 4 = Often (once a week), and 5 = Very Often (more than once a week).

### 2.3. Data Collection Procedure

Participants were recruited via social media platforms and communication channels of pharmacist associations. The survey link was shared to reach a broad audience. Completing the survey took approximately 15–20 min. Data were collected anonymously, and analysis was conducted exclusively for scientific purposes.

### 2.4. Data Analysis

The collected data were coded and analysed using Statistica v13 statistical software. Due to the unequal distribution of groups, results were presented descriptively. Missing data were considered in the analysis and reported in the tables as missing cases. Results for the combined sample are reported as “respondents”; subgroup analyses are reported separately as “pharmacists” and “pharmacy students.”

### 2.5. Ethical Approval

The study received ethical approval from the Research Ethics Board at the Children’s Hospital of Eastern Ontario (CHEO) (REB Protocol No: 20/63X).

## 3. Results

### 3.1. Characteristics of the Sample

This study comprised 68% women and 32% men. The mean age of the respondents was 34.4 years. 93% of the participants were pharmacists and 7% were pharmacy students. The mean length of professional practice was 9.7 years. The vast majority of the respondents (76.4%) work in pharmacies, mainly in cities (Table 1).

### 3.2. Attitudes and Opinions About Medical Cannabis Use

Nearly half of the respondents (48.9%) state that cannabis should be used exclusively for medical purposes, and 47.6% for medical and recreational purposes.

Moving from general attitudes to perceived clinical indications, respondents most often endorsed benefits in adults for cancer (45.5%), difficult-to-treat pain (42.7%), multiple sclerosis (36.5%), epilepsy (33.4%), and nausea (32.9%). For children and adolescents, the most frequently endorsed indications were cancer (38.2%), epilepsy (35.5%), difficult-to-treat pain (33.2%), multiple sclerosis (26.8%), and nausea (24.6%). These results are summarised in Table 2 and Table 3.

**Figure 1:**
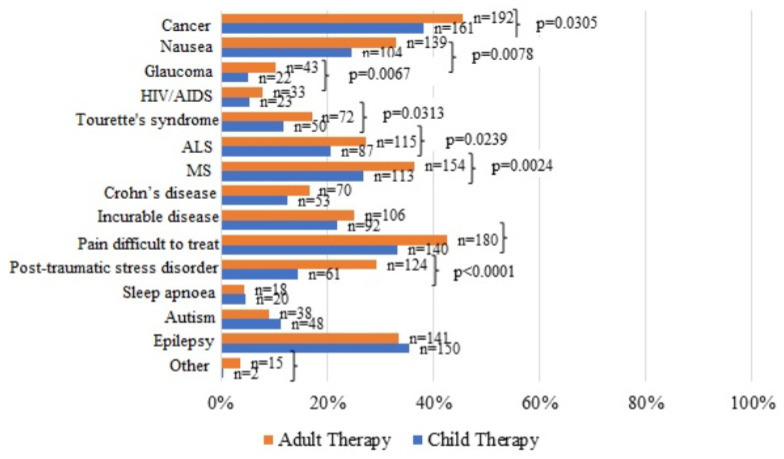
The percentage of responses from the pharmacists and pharmacy students who believe that medical cannabis has a place in medical treatment of various pre-specified indications.

Responses on indications differed between adults and children. Across most conditions, respondents were more likely to endorse use in adults than in children. The largest adult–child gaps concerned post-traumatic stress disorder, pain, nausea, glaucoma, multiple sclerosis, cancers, and Tourette syndrome. Conversely, respondents were relatively more open to paediatric use for autism, epilepsy, and sleep apnoea; however, these differences were not statistically significant (Figure 1).

The question posed was: *“Which indications or symptoms do you think cannabis may be helpful for?”* The participants were allowed to choose multiple indications, and the question was posed separately for adults and children. A total of 64.4% of the respondents strongly agree with the statement that cannabis helps in treating adults, and 39.5% strongly agree that it facilitates children and adolescents treatment (Table 4).

The vast majority of the respondents agree that the benefits of medical cannabis outweigh its risks in adults (60.8%—“I strongly agree”, and 28%—“I agree”). These percentages are slightly lower in the case of children and adolescents (29.4%—“I strongly agree”, and 35.1%—“I agree”), yet most respondents still think that the benefits of medical cannabis outweigh its risks.

Every fourth respondent is afraid that patients who apply for medical cannabis can use it for recreational purposes (16.5% of the respondents agree, and 10.4% strongly agree with this statement). In total, approximately 12% of the respondents are of the opinion that medical cannabis can lead to taking other recreational drugs. Similarly, about 12% of the respondents state that providing advice on the use of medical cannabis can result in legal consequences (Table 5).

Approximately 10% of respondents believed that advising on OTC cannabis products could carry legal consequences, and 14% expressed the same concern regarding dispensing medical cannabis. More than half of the respondents are of the opinion that medical cannabis should only be available in licenced pharmacies (39.7% strongly agree, and 13.5% agree with this statement). Over 67% of the respondents indicate that they feel comfortable talking to patients about medical cannabis use, and a similar percentage feel comfortable talking to patients about OTC cannabis products (Table 6).

Half of the pharmacists and pharmacy students agree with the statement that most people using medical cannabis need pharmacist intervention, and approximately 45% are of the opinion that these intervention are also necessary when taking OTC cannabis products. Nearly 54% of the respondents agree or strongly agree that people who take medical cannabis and additionally suffer from other diseases, should be treated only by specialists in medical cannabis, while 34% think that this also concerns OTC cannabis. A total of 25.1% of the respondents strongly agree and 19.8% agree that they feel comfortable providing advice and answering patient questions regarding the effectiveness of medical cannabis (indications, dosage, types, routes of administration, dosing regimen, monitoring) (Table 7).

A similar percentage of the respondents (45% for “I strongly agree” and “I agree”) indicate that they feel comfortable providing advice and answering patient questions regarding the safety of medical cannabis (adverse effects, drug interactions, counterindications).

Over half of the respondents report that they seldom (less than twice a month) provide advice on medical cannabis or OTC cannabis products. More than 90% of the respondents state that since medical cannabis was legalised, they have seldom administered it to patients (on the basis of a medical order). Nearly all of the respondents (97.8%) report that this legalisation has not changed the way cannabis is perceived (Table 8).

## 4. Discussion

This study shows that 48.9% of respondents favoured medical-only use and 47.6% supported both medical and recreational use (following legalisation), indicating generally favourable yet heterogeneous attitudes toward the therapeutic use of cannabis. Analysis between countries of the availability and possibility of medical use of marijuana shows a significant disharmony between regulations [22]. The pioneers in this respect were the USA and Canada. Currently, in Europe, legal medical cannabis is available, e.g., in the Czech Republic, France, Finland, Germany, Greece, Italy, the Netherlands, Switzerland, and Great Britain [23]. The diseases for which medical cannabis can be used also vary [24]. The most common use of medical cannabis is chronic pain [5]. A report by the National Academies of Sciences, Engineering, and Medicine (NASEM) indicates substantial evidence that cannabis is effective for the treatment of pain, chemotherapy-induced nausea and vomiting, as well as for spasticity in multiple sclerosis. According to NASEM, there is moderate evidence that marijuana is effective for the treatment of sleep disorders, yet there is not sufficient evidence that confirms the impact of cannabis on appetite increase, Tourette syndrome, fear, post-traumatic stress disorder, cancer, irritable bowel syndrome, epilepsy, and various neurodegenerative disorders [13]. Our study aligns partly with these assessments: respondents most frequently indicated cancers, pain that is difficult to treat, multiple sclerosis, epilepsy, and nausea as potential indications.

A large proportion of respondents perceived medical cannabis as effective in adults, and many also reported perceived benefits in children and adolescents; overall, pharmacists and pharmacy students stated that the benefits of medical cannabis outweigh its potential risks. To date, several studies on medical students’ knowledge of the medical use of cannabis have been carried out in Poland. In a study by Mazur R. et al., the majority of students (72%) were of the opinion that cannabis has medicinal properties, and the most frequent indication for medical cannabis was drug-resistant epilepsy; nearly all respondents (92.4%) supported legality for indicated patients, and most favoured prescription-only availability (59.1%) [25]. Other Polish studies indicate that students do not receive sufficient information on the therapeutic use of cannabis and find it difficult to state specific medical uses, most commonly listing cancer, pain, MS, and depression [24]. Following legalisation, substantial knowledge gaps persisted: the correctness of responses regarding medical uses was 24.06%, with “I don’t know” most frequent, underscoring the need to strengthen education to support appropriate patient management [26].

In our study, over 66% of respondents felt comfortable talking to patients about medical cannabis, whereas 46% felt comfortable providing detailed advice about effectiveness. Prior research shows that increased knowledge reduces fears and increases openness to counselling about medical cannabis [27]. Studies from other countries indicate that patients frequently ask clinicians about medical cannabis: 25–95% of doctors are asked, and depending on experience and specialty, 10–95% report being able to answer [28,29,30]. In our sample, this appears lower: most respondents reported providing advice less than twice a month, and over 90% stated that since legalisation they have seldom administered medical cannabis to patients, which is consistent with its limited integration into routine practice.

Although our results suggest that about half of pharmacists feel comfortable advising on medical cannabis, studies from Israel indicate that nearly 80% of doctors feel uncomfortable, primarily due to perceived insufficient knowledge [31]. Canadian studies likewise point to gaps related to constructing effective treatment plans and assessing benefit–risk profiles [32]. Consistent with this, more experienced or better-informed clinicians tend to be more convinced about advantages and less concerned about negative effects [33]. A review of European studies shows generally favourable attitudes toward medical cannabis among healthcare workers, coupled with a recurrent request for additional training [34]. Among pharmacy students in the United States, knowledge levels were low and many felt unprepared to dispense medical cannabis, further highlighting educational needs [35]. Similar patterns were observed among primary care providers—many perceived potential benefits (e.g., for cancer, incurable disease, pain), yet uncertainty about effectiveness was common and nonresponse rates were high [36], findings echoed in Norway [37].

In this context, the pharmacist’s role is particularly important: beyond dispensing, pharmacists provide counselling and often monitor treatment [38]. In our study, over half of respondents believed that medical cannabis use requires pharmacist intervention, and many indicated that patients with comorbidities should be managed by specialists in medical cannabis. Nonetheless, even in early-adopting settings such as California, pharmacists reported insufficient access to credible information and uncertainty about where to find it [39]. Outside Europe and North America, findings from Lebanon show that roughly half of pharmacists demonstrate good knowledge of uses, side effects, and interactions, while 79.4% support strictly defined medical use; knowledge was related to experience, residence, and beliefs [40].

Dosing and safety further underscore pharmacists’ value, as determining a safe and effective regimen requires solid pharmacological knowledge [15]. Use in children is especially challenging: younger age may be associated with higher risks of dependency or adverse effects [41]. Accordingly, paediatric use should be limited to selected indications with carefully planned and monitored treatment [42]. Overall, increasing education on medical cannabis among healthcare professionals is likely to improve standards of care; knowledge appears to be the strongest determinant of counselling comfort for specific conditions [32]. Such initiatives are essential to enable healthcare professionals to advise and educate patients effectively on condition-specific use [43].

Our results align with prior surveys showing generally favourable attitudes toward medical cannabis among pharmacists but uneven confidence in detailed counselling [15,32,34,35,36,37,38,39,40]. Consistent with other studies, respondents supported therapeutic use yet reported knowledge gaps that constrain practice [15,34,38,39,40]. Unlike settings with more mature programmes, Polish pharmacists reported a much lower frequency of counselling/dispensing and greater concern about legal repercussions, reflecting our partial-implementation context [38,39,40]. We also extend earlier work by disaggregating adult versus paediatric indications and by quantifying comfort with indication- and safety-specific counselling domains, which are infrequently reported in pharmacist surveys [34,35,36].

### Limitations

This study has several limitations. Primarily, due to the lack of a standardised questionnaire, we created our own survey, which was distributed online, introducing potential bias related to sampling. Nearly half of the respondents did not complete the questionnaire, which is a significant limitation. Possible explanations include the 15–20 min completion time, the perceived irrelevance of some questions, or breaks from work. Therefore, we provide the denominators for each question and note the risk of nonresponse bias. We also acknowledge that sampling bias may affect the prevalence estimates and some associations (internal validity) and limit the generalisability of the results (external validity). Furthermore, despite the initial assumption that the beliefs and attitudes of pharmacists and pharmacy students regarding medical marijuana use should be compared, such a comparison was not possible due to significant differences in the number of respondents between the two groups.

## 5. Conclusions

Pharmacists in Poland generally support the use of cannabis for medical purposes in addressing specific health conditions in both adults and children, with many acknowledging that the benefits of medical cannabis outweigh its potential risks. While a majority feel confident discussing the medical use of cannabis with patients, fewer express comfort in providing detailed advice or guidance on its use.

The findings highlight a critical need to enhance education and training for pharmacists and pharmacy students regarding the therapeutic applications of medical cannabis. This can be achieved through comprehensive research into the clinical efficacy of cannabis for specific conditions, its long-term side effects, and the knowledge and attitudes of healthcare professionals toward its use. Such studies should explore differences across professional groups and consider demographic variables to ensure tailored and effective educational interventions. Expanding evidence-based knowledge in these areas will help equip pharmacists with the tools necessary to support safe and informed patient care.

## Figures and Tables

**Table 1:** Characteristics of the pharmacists and pharmacy students participating in the survey (N = 422).

Variable	Poland	
	N (%)/Mean (SD)	Sum Total
**Sex**		294 *
Male	94 (32.0%)	
Female	200 (68.0%)	
**Age**	34.4 (9.8)	262 *
**Pharmacy Status**		301 *
Pharmacist	280 (93.0%)	
Pharmacy Student	21 (7.0%)	
**Degree**		
Master’s Degree in Pharmacy	254 (60.2%)	
Doctor of Philosophy Degree	12 (2.8%)	
Professor in Pharmacy	3 (0.7%)	
Hospital Pharmacy Residency	8 (1.9%)	
Industrial Pharmacy Residency	3 (0.7%)	
Other	19 (4.5%)	
Not Applicable	6 (1.4%)	
Prefer not to say	4 (0.9%)	
**Practice (years)**	9.7 (9.6)	281 *
**Practice Setting**		288 *
Industry	13 (4.5%)	
Pharmacy	220 (76.4%)	
Pharmaceutical wholesalers	8 (2.8%)	
Government	2 (0.7%)	
Military	2 (0.7%)	
Hospital	15 (5.2%)	
Academia	15 (5.2%)	
Other	13 (4.5%)	
**Practice Location**		282 *
Rural	18 (6.4%)	
Urban	264 (93.6%)	

**Note:** * Represents unfilled values in the survey (missing cases).

**Table 2:** The percentage of responses from the pharmacists and pharmacy students on their worries and concerns regarding potential repercussions associated with dispensing cannabis and providing advice (N = 231/422).

Variable	Poland
	N (%)
**I think that cannabis:**	
Only medical purposes	113 (48.9%)
Medical and recreational purposes, legalisation	110 (47.6%)
Medical and recreational purposes, without legalisation	8 (3.5%)
**What conditions can cannabis help treat in adults**	
Cancer	192 (45.5%)
Nausea	139 (32.9%)
Glaucoma	43 (10.2%)
HIV/AIDS	33 (7.8%)
Tourette syndrome	72 (17.1%)
ALS	115 (27.3%)
MS	154 (36.5%)
Crohn’s disease	70 (16.6%)
Incurable disease	106 (25.1%)
Pain difficult to treat	180 (42.7%)
Post-traumatic stress disorder	124 (29.4%)
Sleep apnoea	18 (4.3%)
Autism	38 (9.0%)
Epilepsy	141 (33.4%)
Other	15 (3.6%)

**Table 3:** The percentage of responses from the pharmacists and pharmacy students on their worries and concerns regarding potential repercussions associated with dispensing cannabis and providing advice (N = 422).

Variable	Poland (*n* = 422)	
	N (%)	Sum Total
**What conditions can cannabis help treat in children and adolescents**		
Cancer	161 (38.2%)	
Nausea	104 (24.6%)	
Glaucoma	22 (5.2%)	
HIV/AIDS	23 (5.5%)	
Tourette syndrome	50 (11.8%)	
ALS	87 (20.6%)	
MS	113 (26.8%)	
Crohn’s disease	53 (12.6%)	
Incurable disease	92 (21.8%)	
Pain difficult to treat	140 (33.2%)	
Post-traumatic stress disorder	61 (14.5%)	
Sleep apnoea	20 (4.7%)	
Autism	48 (11.4%)	
Epilepsy	150 (35.5%)	
Other	2 (0.5%)	

**Table 4:** The percentage of responses from the pharmacists and pharmacy students on their worries and concerns regarding potential repercussions associated with dispensing cannabis and providing advice (N = 233/422).

Survey Question	-1- Strongly Disagree	-2-Disagree	-3- Neither Disagree nor Agree	-4- Agree	-5- Strongly Agree
**I think that cannabis helps in treating adults**					
(233)	4 (1.7%)	6 (2.6%)	12 (5.2%)	61 (26.2%)	150 (64.4%)
**I think that cannabis helps in treating children and adolescents**					
(233)	9 (3.9%)	14 (6.0%)	50 (21.5%)	68 (29.2%)	92 (39.5%)

**Table 5:** The percentage of responses from the pharmacists and pharmacy students on their worries and concerns regarding potential repercussions associated with dispensing cannabis and providing advice (N = 230–232/422).

Survey Question	-1- Strongly Disagree	-2-Disagree	-3- Neither Disagree nor Agree	-4- Agree	-5- Strongly Agree
**I think that the benefits of medical cannabis outweigh the risks in the case of adults:**					
(232)	6 (2.6%)	5 (2.2%)	15 (6.5%)	65 (28%)	141 (60.8%)
**I think that the benefits of medical cannabis outweigh the risks in the case of children and adolescents:**					
(231)	12 (5.2%)	14 (6.1%)	56 (24.2%)	81 (35.1%)	68 (29.4%)
**I’m afraid that patients who apply for medical cannabis can use it for recreational purposes:**					
(230)	65 (28.3%)	58 (25.2%)	45 (19.6%)	38 (16.5%)	24 (10.4%)
**I’m afraid that medical cannabis can lead to taking other recreational drugs:**					
(230)	98 (42.6%)	58 (25.2%)	45 (19.6%)	18 (7.8%)	11 (4.8%)
**I’m afraid that providing advice on the use of medical cannabis can result in legal consequences:**					
(230)	120 (52.2%)	42 (18.3%)	40 (17.4%)	17 (7.4%)	11 (4.8%)

**Table 6:** The percentage of responses from the pharmacists and pharmacy students on their worries and concerns regarding potential repercussions associated with dispensing cannabis and providing advice (N = 226–229/422).

Survey Question	-1- Strongly Disagree	-2-Disagree	-3- Neither Disagree nor Agree	-4- Agree	-5- Strongly Agree
**I’m afraid that providing advice on the use of OTC cannabis products can result in legal consequences:**					
(228)	139 (61%)	39 (17.1%)	28 (12.3%)	11 (4.8%)	11 (4.8%)
**I’m afraid that dispensing medical cannabis to patients can result in legal consequences:**					
(229)	129 (56.3%)	39 (17.0%)	29 (12.7%)	18 (7.9%)	14 (6.1%)
**I’m afraid that medical cannabis should be available only in licensed pharmacies** **:**					
(229)	58 (25.3%)	23 (10%)	26 (11.4%)	31 (13.5%)	91 (39.7%)
**I feel comfortable talking to patients about medical cannabis use** **:**					
(228)	11 (4.8%)	19 (8.3%)	45 (19.7%)	60 (26.3%)	93 (40.8%)
**I feel comfortable talking to patients about OTC cannabis products** **:**					
(226)	15 (6.6%)	13 (5.8%)	48 (21.2%)	63 (27.9%)	87 (38.5%)

**Table 7:** The percentage of responses from the pharmacists and pharmacy students on their worries and concerns regarding potential repercussions associated with dispensing cannabis and providing advice (N = 227–228/232).

Survey Question	-1- Strongly Disagree	-2-Disagree	-3- Neither Disagree nor Agree	-4- Agree	-5- Strongly Agree
**I think that most people using medical cannabis need pharmacist intervention** **:**					
(227)	14 (6.2%)	26 (11.5%)	70 (30.8%)	64 (28.2%)	53 (23.3%)
**I think that most people using OTC cannabis products need pharmacist intervention:**					
(227)	19 (8.4%)	40 (17.6%)	66 (29.1%)	64 (28.2%)	38 (16.7%)
**I think that people who take medical cannabis and additionally suffer from other diseases should be treated only by specialists in medical cannabis** **:**					
(228)	20 (8.8%)	38 (16.7%)	47 (20.6%)	52 (22.8%)	71 (31.1%)
**I think that people who take OTC cannabis productsand additionally suffer from other diseases, should be treated only by specialists in medical cannabis:**					
(228)	41 (18.0%)	59 (25.9%)	50 (21.9%)	42 (18.4%)	36 (15.8%)
**I feel comfortable providing advice and answering patient questions regarding the effectiveness of medical cannabis (indications, dosage, types, routes of administration, dosing regimen, monitoring)**					
(227)	27 (11.9%)	39 (17.2%)	59 (26.0%)	45 (19.8%)	57 (25.1%)

**Table 8:** The percentage of responses from the pharmacists and pharmacy students on their worries and concerns regarding potential repercussions associated with dispensing cannabis and providing advice (N = 224–230/422).

Survey Question	-1- Strongly Disagree/Never	-2-Disagree/Rarely	-3- Neither Disagree nor Agree/Sometimes	-4- Agree/ Often	-5- Strongly Agree/Very Often
**I feel comfortable providing advice and answering patient questions regarding the safety of medical cannabis (adverse effects, drug interactions, counterindications)**					
(224)	24 (10.7%)	41 (18.3%)	55 (24.6%)	49 (21.9%)	55 (24.6%)
**How often do you provide advice on medical cannabis?**					
(226)	73 (32.3%)	118 (52.2%)	22 (9.7%)	6 (2.7%)	7 (3.1%)
**How often do you provide advice on OTC cannabis products?**					
(227)	50 (22.0%)	124 (54.6%)	39 (17.2%)	9 (4.0%)	5 (2.2%)
**How often have you personally used medical cannabis since it was legalised?**					
(230)	15 (6.5%)	208 (90.4%)	7 (3.0%)	0 (0.0%)	0 (0.0%)
**To what extent has your way of perceiving cannabis changed since it was legalised** **?**					
(229)	99 (43.2%)	125 (54.6%)	5 (2.2%)	0 (0.0%)	0 (0.0%)

## Data Availability

All data are available from the corresponding author.
